# Optimization of Collagen Chemical Crosslinking to Restore Biocompatibility of Tissue-Engineered Scaffolds

**DOI:** 10.3390/pharmaceutics13060832

**Published:** 2021-06-03

**Authors:** Mohammad Mirazul Islam, Dina B. AbuSamra, Alexandru Chivu, Pablo Argüeso, Claes H. Dohlman, Hirak K. Patra, James Chodosh, Miguel González-Andrades

**Affiliations:** 1 Department of Ophthalmology, Massachusetts Eye and Ear and Schepens Eye Research Institute, Harvard Medical School, Boston, MA 02114, USA; mohammad_islam@meei.harvard.edu (M.M.I.); dina_abusamra@meei.harvard.edu (D.B.A.); Pablo_Argueso@MEEI.HARVARD.EDU (P.A.); claes_dohlman@meei.harvard.edu (C.H.D.); james_chodosh@meei.harvard.edu (J.C.); 2 Department of Surgical Biotechnology, University College London, London NW3 2PF, UK; a.chivu@ucl.ac.uk (A.C.); hirak.patra@ucl.ac.uk (H.K.P.); 3 Maimonides Biomedical Research Institute of Cordoba (IMIBIC), Department of Ophthalmology, Reina Sofia University Hospital and University of Cordoba, 14004 Cordoba, Spain

**Keywords:** cornea, collagen, double-crosslinking, carbodiimide, glutaraldehyde, sodium metabisulfite, sodium borohydride, EDC/NHS

## Abstract

Collagen scaffolds, one of the most used biomaterials in corneal tissue engineering, are frequently crosslinked to improve mechanical properties, enzyme tolerance, and thermal stability. Crosslinkers such as 1-ethyl-3-(3-dimethylaminopropyl) carbodiimide hydrochloride (EDC) are compatible with tissues but provide low crosslinking density and reduced mechanical properties. Conversely, crosslinkers such as glutaraldehyde (GTA) can generate mechanically more robust scaffolds; however, they can also induce greater toxicity. Herein, we evaluated the effectivity of double-crosslinking with both EDC and GTA together with the capability of sodium metabisulfite (SM) and sodium borohydride (SB) to neutralize the toxicity and restore biocompatibility after crosslinking. The EDC-crosslinked collagen scaffolds were treated with different concentrations of GTA. To neutralize the free unreacted aldehyde groups, scaffolds were treated with SM or SB. The chemistry involved in these reactions together with the mechanical and functional properties of the collagen scaffolds was evaluated. The viability of the cells grown on the scaffolds was studied using different corneal cell types. The effect of each type of scaffold treatment on human monocyte differentiation was evaluated. One-way ANOVA was used for statistical analysis. The addition of GTA as a double-crosslinking agent significantly improved the mechanical properties and enzymatic stability of the EDC crosslinked collagen scaffold. GTA decreased cell biocompatibility but this effect was reversed by treatment with SB or SM. These agents did not affect the mechanical properties, enzymatic stability, or transparency of the double-crosslinked scaffold. Contact of monocytes with the different scaffolds did not trigger their differentiation into activated macrophages. Our results demonstrate that GTA improves the mechanical properties of EDC crosslinked scaffolds in a dose-dependent manner, and that subsequent treatment with SB or SM partially restores biocompatibility. This novel manufacturing approach would facilitate the translation of collagen-based artificial corneas to the clinical setting.

## 1. Introduction

Data from the World Health Organization (WHO) indicate that the current transplantation rate of 100,800 solid organs per year fulfills less than 10% of the global demand [1]. Therefore, any advancement in the development of new biomaterials holds tremendous potential to fill the gap between the supply and demand of donated organs. Engineered organ development is based on the selection of appropriate biomaterials, which usually requires polymerization processes to form apposite structure of the target organ. Natural biomaterials, such as collagen, show superior biocompatibility but lack optimal mechanical properties. Despite that, collagen-based biomaterials are widely used for tissue engineering because of their excellent properties for creating regenerative cell-free scaffolds for tissue engineering as seen in early clinical evaluations [2,3,4]. The clinical success of collagen scaffolds is based on their excellent biocompatibility. However, bioactivity is hindered mostly by poor mechanical behavior and a high vulnerability to enzymatic digestion [5]. These weaknesses make collagen scaffolds unsuitable for transplantation in patients with severe inflammatory disease conditions in which high levels of host proteolytic enzymes are present.

Crosslinkers are commonly used to produce collagen-based scaffolds with physicochemical properties similar to healthy tissues and organs, including mechanical properties, enzyme tolerance capacity, and thermal stability. Collagen can be crosslinked with different strategies that include chemical [6], physical [7], and enzymatic [8] crosslinking. One of the most commonly used is the zero-length chemical crosslinking using 1-ethyl-3-(3-dimethylaminopropyl) carbodiimide hydrochloride (EDC) and N-hydroxysuccinimide (NHS), which have been proven effective for crosslinking of collagen fibres [9]. This approach results in a lower cross-linking density than other chemical (e.g., GTA, formaldehyde) and physical crosslinking methods (e.g., ionizing irradiation or UV light, dehydrothermal, pressure), but has been shown to have promising biofunctional outcomes [3], unlike more hostile crosslinking techniques that are associated with inflammatory responses and worse biological outcomes [10]. Crosslinking using EDC involves the activation of carboxylic acid groups of collagen to give o-acylisourea groups, which form crosslinks after reaction with free amine groups of the collagen and finally formed amide bonds [11]. In this process, EDC does not incorporate into the crosslinked structure. Because of that, this type of crosslinking is called zero-length crosslinking; however, there are non-zero-length crosslinkers that get incorporated into the crosslinked structure with a series of chemical reactions [12]. Although EDC/NHS forms rigid zero-length crosslinking of the collagen, the collagen hydrogel still remain susceptible to collagenase degradation and the mechanical strength of crosslinked collagen is not satisfactory [5].

To address the lack of enzymatic stability and mechanical strength of EDC-crosslinked collagen scaffolds, several research groups have proposed other crosslinkers for collagen, such as glutaraldehyde (GTA) [13]. GTA is a non-zero-length crosslinker that generates a more rigid biomacromolecular network, associated with the incorporation of covalent bonds (Schiff bases) [14] between polypeptide chains of the collagen. GTA has been used for clinical applications, not only as a crosslinker but also as a sterilizing agent, including the fabrication of bioprostheses for human implantation [15]. However, other medical applications of GTA crosslinking are generally restricted as chemical modification of biomaterials by GTA may pose a significant risk for toxicity [16,17]. Thus, there is a need to optimize the GTA-based crosslinking in order to maximize the improvement of physicochemical properties of artificial tissues and organs while minimizing its toxic effect.

The balance between mechanical resistance, including enzymatic stability, and cytotoxicity can be achieved by two main approaches. On the one hand, as we have shown previously, different crosslinkers can be combined to crosslink collagen in order to acquire the beneficial effect of each crosslinker [18]. GTA has not been previously used in combination with other more biocompatible crosslinkers with the direct purpose of reducing the exposure to GTA in order to decrease GTA-induced cytotoxicity. However, as an evaluation of physical properties of collagen membranes, it was shown that GTA can increase the crosslinking density of a previously crosslinked matrix using dimethyl suberimidate as first crosslinker [19]. On the other hand, different research groups have shown that the aldehyde groups introduced in the crosslinked biopolymers with GTA can be masked with different chemical treatments (i.e., glycine) to reduce the cytotoxicity of glutaraldehyde [20].

Therefore, we aimed to optimize double-crosslinking of collagen scaffolds using EDC/NHS and GTA, including masking chemical treatments in order to manufacture mechanically and enzymatically strong implants while reducing the cytotoxic effect of the crosslinkers. We focused on the corneal application because of the high requirements in terms of mechanical and optical behavior, in addition to biocompatibility. Herein, we crosslinked collagen with EDC and post-treated with GTA, followed by different neutralizing chemical agents (i.e., sodium metabisulfite (SM) and sodium borohydride (SB)) to restore the biocompatibility of the implants. Our result demonstrated that GTA improves the mechanical properties of the EDC crosslinked implants in a dose-dependent manner, and a final masking of the free aldehyde group on the scaffold with SM or SB can restore biocompatibility.

## 2. Materials and Method

Type I porcine atelocollagen was purchased from Nippon Meat Packers Inc. (Tokyo, Japan). All reagents were of analytical grade and used as received. All the chemicals were purchased from Sigma-Aldrich (St. Louis, MO, USA) if not mentioned otherwise. 

### 2.1. Fabrication of Collagen Hydrogel

Hydrogel encompassed 10% (wt/wt) collagen was made following previously described protocol [18]. Collagen solution was buffered with 150 μL of 0.63 M 2-(N-Morpholino)ethanesulfonic acid, 4-Morpholineethanesulfonic acid monohydrate (MES) buffer in a syringe mixing system. Then, the collagen solution was adjusted to pH 5 with 2.0 M aqueous NaOH. Calculated volumes of aqueous solutions of 1-ethyl-3-(3-dimethylaminopropyl) carbodiimide (EDC) and its co-reagent N-hydroxysuccinimide (NHS), both at 10% (wt/vol), were added to the collagen solution (EDC: Collagen-NH_2_ (mol:mol) = 0.7:1 and EDC:NHS (mol:mol) = 2:1). Every addition was followed by thorough mixing. The final mixed solution was immediately dispersed onto a glass plate to a thickness of 500 µm, similar to the human cornea. The hydrogels were cured at 100% humidity at room temperature for 16 h and then at 37 °C for 5 h. After demolding, hydrogels were washed thoroughly with phosphate-buffered saline (PBS).

### 2.2. Double-Crosslinking with GTA

Different molar concentrations of GTA were used to double-crosslink the collagen hydrogels. The molar ratio of GTA:Collagen-NH_2_ was set at x:1, where x was gradually increased to 0.018, 0.18, 0.45, 0.9, and 2.7. Depending on x, hydrogels were named as 1, 2, 3, 4, and 5, respectively. Three 6 mm diameter hydrogels were placed in a glass vial with 1 mL PBS containing a specific amount of GTA and the vial was kept under continuous shaking at 150 rpm for 4 h. After GTA crosslinking, the hydrogels were washed rigorously with PBS overnight at room temperature. We used neutral pH conditions for GTA crosslinking. At acidic conditions, GTA crosslinking is usually slow and gives materials of lower thermal stability [21].

### 2.3. Masking Unreacted Aldehyde Groups

Double-crosslinked hydrogels were treated with different chemicals to mask the unreacted aldehyde group from the hydrogel to reduce cytotoxicity. Sodium metabisulfite 1% (wt/vol) (SM) and Sodium borohydride 1% (wt/vol) (SB) were independently used for 1 h and 10 min, respectively, to treat the hydrogels under constant shaking at room temperature. We also tested the efficacy of 200 mM glycine, 200 nM lysine, 10% citric acid, 200 mg/mL NaOH and 4 M NaCl for 1.5 h to mask unreacted aldehyde groups of the double-crosslinked hydrogel. However, due to their inability to prevent cytotoxicity, these groups were taken out from the further studies, continuing the evaluation of SM and SB.

### 2.4. Optical Transmission

The optical transmissions of the hydrogels were examined by a UV-Vis spectrometer (Molecular Devices SpectraMax 384 Plus Microplate Reader, Molecular Devices; San Jose, CA, USA). Six mm diameter trephined discs of the hydrogels were placed in individual wells of a 96-well quartz microplate, and their optical transmittance was recorded from 200–800 nm in quartz microplate at 1 nm wavelength increments. The transmittance of the samples was corrected with blank water media and the mean transmittance (%) for each group was calculated and plotted as a function of wavelength.

### 2.5. In Vitro Biodegradation

The resistance of the hydrogels against collagenase digestion was determined as we previously described [22]. In brief, hydrogels were placed in a vial containing 5 U/mL collagenase from *Clostridium histolyticum* (Sigma-Aldrich, St. Louis, MO, USA) in 0.1 M Tris-HCl (pH 7.4) and 5 mM CaCl_2_ at 37 °C. The collagenase solution was replenished every 8 h and the percent residual mass of the sample was measured at different time points after removing the hydrogels from the solution and gently blotted on the filter paper to remove the surface water.

### 2.6. Mechanical Characterization

Mechanical characterization of the hydrogels was conducted using a mechanical tester (Mark-10 ESM 303, Copiague, NY, USA). To measure the compressive modulus, cylindrical samples (diameter = 6.0 mm, and thickness = 0.5 µm) were placed in the mechanical tester and measurement was performed with crosshead speed of 0.5 mm/min. The compressive stress was recorded as a function of the strain. Load displacement extracted data were translated to engineering stress–strain data by incorporating the cross-sectional areas and the original thickness of the hydrogels. The obtained stress/strain curve was used to extract the compressive modulus of each hydrogel.

### 2.7. Water Content Measurement

The water content of hydrogels was determined to ensure uniformity using previously published protocol [3] with modifications. In brief, hydrated hydrogels were removed from PBS; the surface was gently blotted dry and then immediately weighed on a microbalance to record the wet weight (W_0_) of the hydrogels. Dry weight of the same hydrogels was obtained by drying the samples at 50 °C until constant mass was achieved (W).

The equilibrated water content of the hydrogels (W_t_) was calculated according to the following equation: W_t_ = (W_0_ − W)/W_0_  ×  100%.

### 2.8. Fourier-Transform Infrared Spectroscopy (FTIR)

FTIR was performed on a Jasco attenuated total reflectance FTIR 4200 spectrometer (ATR FT/IR-4200, Jasco, Tokyo, Japan), averaging 30 scans between 4000 cm^−1^ and 600 cm^−1^, at a resolution of 2 cm^−1^. The measurements were performed on the samples in hydrated form, as well as after drying in a vacuum desiccator for 24 h.

### 2.9. Contact Angle Measurement

The surface hydrophilicity of different hydrogel samples was studied by contact angle measurements. A drop of 3 μL dH_2_O was deposited onto each hydrogel by a micro-syringe and images were taken with Dino-light digital microscope (AnMo Electronics Corporation, Hsinchu, Taiwan). The contact angle was measured with ImageJ (U.S. National Institutes of Health, Bethesda, MD, USA).

### 2.10. In Vitro Biocompatibility

Human corneal cells were used to evaluate the biomaterials as the proposed modification could be applied for the improvement of collagen based artificial cornea development. The cornea is a non-compartmented, solid, transparent organ that is comprised mainly of three distinct cell types. Each of these corneal cell populations behaves differently in response to the specific properties of the biomaterial. For that reason, we specifically checked all three principal cell types in conjugation with the modified double-crosslinked hydrogels.

#### 2.10.1. Human Corneal Epithelial Cells (HCEC)

The biocompatibility of the hydrogels was tested using SV40-immortalized HCEC, kindly provided by Professor May Griffith, as previously reported [23]. The hydrogels were cut into 6 mm diameter segments and 5000 HCEC were seeded on top of each for culture with DMEM/Ham’s F-12 media (Corning, Manassas, VA, USA) supplemented with 10% Newborn Calf Serum (NCS) (HyClone, Logan, UT, USA), 10 ng/mL epidermal growth factor (EGF) (Life Technologies, Frederick, MD, USA), 5 ug/mL Human Insulin (Sigma-Aldrich, St. Louis, MO, USA) and 1% penicillin/streptomycin (Gibco, Life Technologies Corporation, Grand Island, NY, USA), at 37 °C and 5% CO_2_. Cells seeded on tissue culture plates (TCP) were used as control. AlamarBlue study was performed at day 1, day 4, and day 7 after cell seeding. At each time point, the tissue culture media was removed and replaced with fresh media (100 μL) containing resazurin sodium salt (0.004% *w*/*v*) and incubated for 4 h. Afterwards, the media (95 μL) were removed from each well and pipetted into a new 96-well plate and read on a BioTek plate reader (Synergy 2, BioTek Instruments; Winooski, VT, USA) at 530/25 nm for excitation and 600/25 nm for emission. At day 7, live/dead staining was performed with a staining kit (Life Technologies Corporation, Carlsbad, CA, USA), where cells were double-stained by calceinacetoxymethyl (Calcein AM) and ethidium homodimer-1 (EthD-1). Images were taken by using a fluorescence microscope (Zeiss Axio Observer Z1, Carl Zeiss Microimaging GmbH, Jena, Germany). The transcellular barrier function of stratified corneal epithelial cells was evaluated with the rose bengal anionic dye (Acros Organics; Morris Plains, NJ, USA) as described previously [24].

#### 2.10.2. Human Corneal Fibroblasts (HCF)

Immortalized human corneal fibroblasts (HCF), kindly donated by Professor James V Jester, were used for evaluating the cellularization potential. Around 5000 HCF were cultured on the top of the hydrogels and cultured with DMEM/Ham’s F-12 media supplemented with 10% fetal bovine serum (FBS) (Life Technologies Corporation, Carlsbad, CA, USA) for seven days at 37 °C in 5% CO_2_. AlamarBlue assay was performed at day 1, 4, and 7 after cell seeding and live/dead staining was performed at day 7.

#### 2.10.3. Human Corneal Endothelial Cells (CEC)

Telomerase immortalized human corneal endothelial cells (CEC), kindly provided by Professor Ula Jurkunas, were cultured on the top of hydrogels. Cells were cultured in Opti-MEM I with Glutamax-I media (Life Technologies Corporation, Carlsbad, CA, USA) supplemented with 8% (*v*/*v*) FBS, 5 ng/mL EGF (EMD Millipore Corporation, Temecula, CA, USA), 0.2 mg/mL calcium chloride (Fisher Scientific Company, Fair Lawn, NJ, USA), 0.8 mg/mL chondroitin sulfate-A (Sigma-Aldrich, St. Louis, MO, USA), 0.25 mg/mL Gentamycin (Life Technologies Corporation), 1% (*v*/*v*) Antibiotic-Antimycotic solution (Life Technologies Corporation), and 0.1 mg/mL bovine pituitary extract (Alfa Aesar, Ward Hill, MA, USA) for 7 days. Approximately 5000 CEC were seeded on the top of the hydrogels and incubated at 37 °C in 5% CO_2_. Media was changed every other day. AlamarBlue assay was performed at day 1, 4, and 7 after cell seeding, and live/dead staining was performed at day 7.

### 2.11. Hydrogel Composition and Influence on Human Adaptive Immunity

Human monocytic THP-1 cells were used to determine the effect of the hydrogels on human adaptive immunity. THP-1 cells were cultured on the hydrogels in RPMI (Gibco) media supplemented with 10% FBS, 1% penicillin/streptomycin and 50 μM β-mercaptoethanol (Gibco) and culture for 6 days at 37 °C in 5% CO_2_. THP-1 cells culture on TCP with or without Lipopolysaccharide (1× LPS, Invitrogen, Carlsbad, CA, USA) were used as controls. Morphological changes of the cells were evaluated by 20× phase contrast images taken at days 2 and 6 with Nikon Eclipse (TS100) microscope. LPS was used as a control to induce THP-1 cell differentiation into macrophage-like cells. The influence of hydrogels on pro-inflammatory macrophage differentiation was evaluated by labeling the cells after culture for 6 days with direct-conjugate antibody against CD86 (pro-inflammatory M1 marker) (Table 1). Data were acquired using a BD LSR II and analyzed using FlowJo software (Becton, Dickinson and Company, Franklin Lakes, NJ, USA).

### 2.12. Statistical Analysis

One-way ANOVA with Tukey post hoc test was performed to compare mechanical and functional characteristic properties. A value of *p* < 0.05 was considered statistically significant. n.s., *, **, ***, and **** represent *p* greater than 0.05, *p* < 0.05, *p* < 0.01, *p* < 0.001, and *p* < 0.0001, respectively. For cell culture study, statistics were not denoted on the graph due to over crowdedness in the figure; however, result elaboration contains the specific statistics data. The GraphPad Prism Software (GraphPad Software, San Diego, CA, USA) was used to analyze the data.

## 3. Results

### 3.1. Collagen Hydrogel

**Figure 1:**
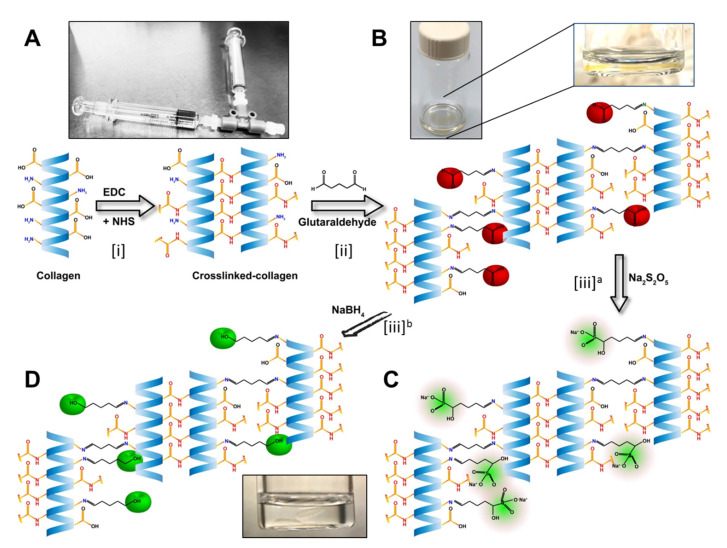
Stepwise illustration of the chemical reactions in the development of the biomaterials. (**A**) Illustration of the EDC/NHS crosslinking of the collagen. The T-piece syringe mixing system was used for making the hydrogels. (**B**) Hydrogels from the previous step crosslinked with GTA, leaving the unreacted aldehyde groups marked as red. Magnified image of a hydrogel in the GTA solution. (**C**) Neutralization of the free aldehyde groups with Sodium metabisulfite (Na_2_S_2_O_5_; in green). Alternatively, (**D**) neutralization of the free aldehyde groups by Sodium borohydride (NaBH_4_) and conversion of aldehyde group to alcohol groups (green).

The hydrogels were developed using collagen as base materials through EDC/NHS crosslinking. The treatments were performed on the base materials and untreated samples served as controls (Figure 1). Post-formulation treatments started with treating hydrogels with different concentrations of GTA solution for producing a mechanically and enzymatically reinforced material suitable for fabrication of the implants.

**Figure 2:**
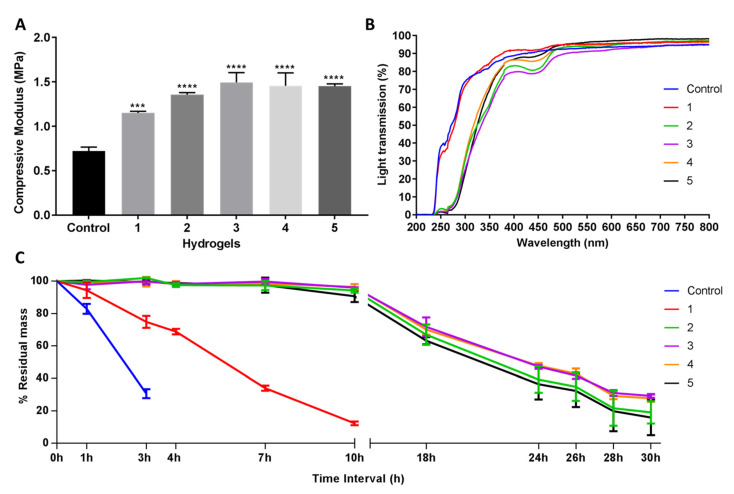
Mechanical and functional assessment of the collagen hydrogels, comparing the control (EDC/NHS crosslinked hydrogel) with double-crosslinked hydrogels with different concentrations of GTA (1, 2, 3, 4, and 5 PFGT hydrogels). (**A**) Compressive modulus was measured to evaluate the mechanical properties of the hydrogels. *** and **** represent *p* < 0.001 and *p* < 0.0001, respectively. (**B**) Optical evaluation of the hydrogels was carried out based on the analysis of light transmission through the different samples, from UV to visual wavelengths. (**C**) Treatment with collagenase was performed and residual mass (presented against time in hours (h)) of the hydrogels was calculated. For all panels, quantitative results were reported as the mean ± S.D. from three independent hydrogels and results were compared between groups.

The compressive modulus was measured to evaluate the mechanical properties of the hydrogel; strong materials exhibit a higher compressive modulus. Post-formulation GTA-treatment (PFGT) significantly increased the mechanical property of EDC-treated hydrogels with respect to the untreated control (non-PFGT) in a dose-dependent manner, reaching a plateau at 1.5MPa. Significant improvement was achieved between 1 and 3 (*p* = 0.0019), 4 (*p* = 0.0048), and 5 (*p* = 0.0055) GTA hydrogels in PFGT groups (Figure 2A). Although GTA concentrations increased, there was no significant difference in mechanical property between 2, 3, 4, and 5 PFGT hydrogels groups.

The UV–Vis spectroscopy revealed that the final PFGT products were transparent (Figure 2B). Transparency to UVB (280–315 nm) was more prominent for control and 1 hydrogels (~70%). UVB transmission was ~30% for all other hydrogels. The PFGT 3 hydrogel formulation more effectively blocked UVA (315–400 nm). The optical evaluation of the hydrogels showed high levels of optical transmission (>80%) in the visible spectrum (between 400 and 800 nm) for the control and 1 hydrogel groups (Figure 2B). Higher doses of GTA led to a decrease of the transmission of visible violet (380–450 nm) and blue (450–485 nm) light. This resulted in color changes after GTA treatment, where yellowing of the hydrogel was observed. Transmission of other visible light (485–800 nm) was similar for all the hydrogels.

Solutions with excess collagenase were used to evaluate the enzymatic stability of the samples in comparison to control non-PFGT hydrogel. Control hydrogels completely degraded within 4 hours (Figure 2C). The lowest concentration of GTA (hydrogel 1) retained a mass of more than 15% after 10 h. All other PFGT groups were stable through 30 h. Approximately 30% residual mass was present for the 3 and 4 PFGT formulations after 30 h collagenase treatment. Like mechanical property, enzymatic stability did not increase further after 3 PFGT hydrogel formulation (Figure 2C).

### 3.2. Masking of Unreacted Aldehyde Groups of GTA

The aldehyde groups introduced in the PFGT hydrogels were quenched by sodium metabisulfite or sodium borohydride, separately, to compare the masking potential of these two chemicals. Based on the mechanical and enzymatic properties, only PFGT 3 hydrogels were used for the aldehyde masking study, in comparison to unmodified PFGT 3 hydrogels. Sodium metabisulfite treated and sodium borohydride treated hydrogels were abbreviated as 3-SM and 3-SB hydrogel, respectively.

**Figure 3:**
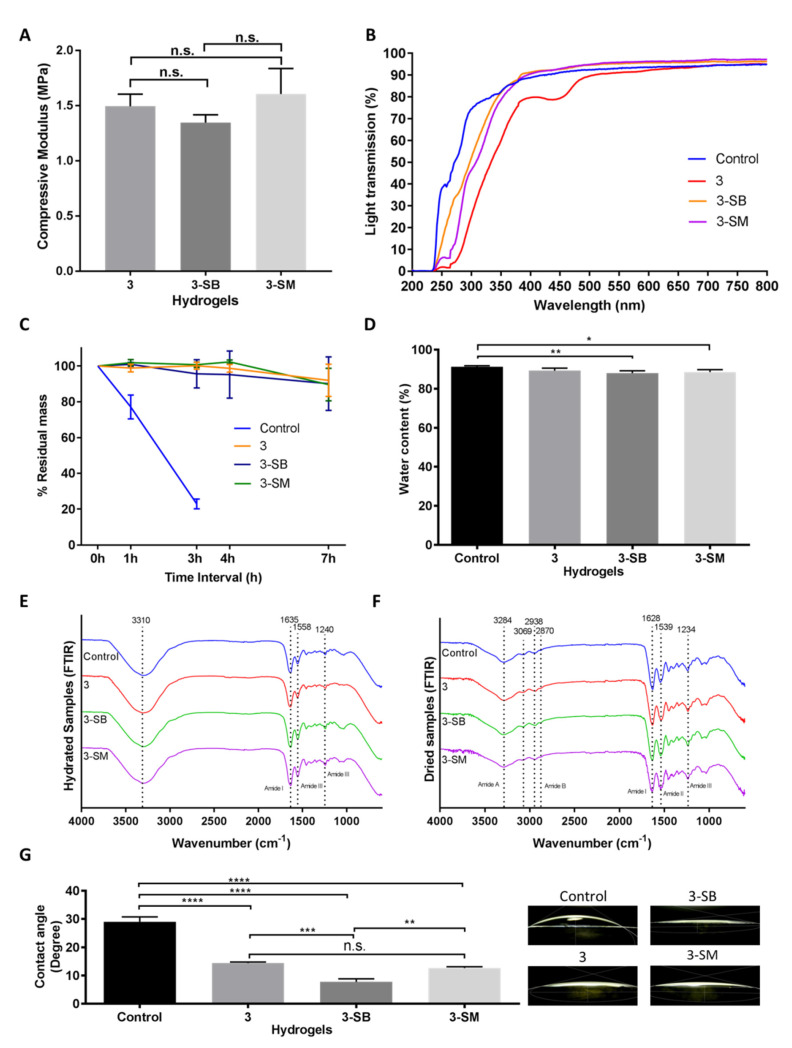
Mechanical and functional assessment of the collagen hydrogels, comparing the control (EDC/NHS) with post-formulation GTA-treatment (PFGT) 3 hydrogels before and after post-crosslinked chemical treatment. Sodium metabisulfite treated and Sodium borohydride treated hydrogels were abbreviated as 3-SM and 3-SB hydrogel, respectively. (**A**) Compressive modulus was measured to evaluate the mechanical properties of the 3 hydrogels. (**B**) Optical evaluation of the hydrogels was carried out based on the analysis of light transmission. (**C**) Collagenase study was performed and residual mass (presented against time in hours (h)) of the hydrogels were calculated. (**D**) Water content measurement (%) among the hydrogels compared with control hydrogels. FTIR spectra of hydrated (**E**) and dried (**F**) hydrogel samples. (**G**) Representative micrographs of water contact angles of different hydrogels with the corresponding contact angle measurement. For all panels, quantitative results were reported as the mean ± S.D. from three independent samples and results compared between groups. n.s., *, **, ***, and **** represent *p* greater than 0.05, *p* < 0.05, *p* < 0.01, *p* < 0.001, and *p* < 0.0001, respectively.

Hydrogels were characterized similarly to evaluate the effect of post-chemical treatment with SM or SB on the PGTA 3 hydrogels. Compressive modulus studies showed that post-chemical treatment of the PGTA hydrogel does not mechanically alter the hydrogel (Figure 3A). The compressive modulus of the hydrogels was 1.5 MPa with non-significant (*p* = 0.1989) differences between hydrogels.

Changes in light transmission were prominent between chemically treated and untreated PFGT 3 hydrogels. Post-crosslinked chemical treatment removes the yellow color (Figure 1) and rendered the hydrogel completely transparent at the visible, violet, and blue light spectrums (Figure 3B). UVA and UVB transmission were also increased but less so compared to the control hydrogel.

The effect of post-chemical treatment on enzymatic stability was also evaluated and examined for 7 h to determine the pattern of the enzymatic degradation, similar to Figure 2C. SB or SM-treated PGTA 3 hydrogels were equally stable to non-treated PFGT 3 hydrogels up to 7 h, and the residual mass was close to the initial weight at the end of each study (Figure 3C). The pattern of degradation was similar to the extended time study previously performed (Figure 2C).

Water content of the control hydrogel was similar to non-treated PFGT 3 hydrogel, although there were significant differences between control and 3-SB and 3-SM hydrogels. However, for all hydrogels, the water content was more than 80% (Figure 3D).

The FTIR spectra of the hydrated gel (Figure 3E) exhibited amide I bands at 1635 cm^−1^ attributed to stretching of C=O bonds of the polypeptide chains, as well as amide II and amide III bands at 1558 cm^−1^ and 1240 cm^−1^, respectively, associated with in-plane N-H bending, C-N stretching, and C-H stretching. The potential amide A and amide B bands were obscured by the presence of a broad band centred at 3310 cm^−1^ characteristic of O-H stretching vibration mode of water. The dried hydrogel samples (Figure 3F) showed similar spectral features to the ones in the hydrated state, with slightly shifted band positions. The FTIR spectra showed amide A bands associated with N-H stretching at 3284 cm^−1^, with shoulders at 3069 cm^−1^ corresponding to sp^2^ C-H stretching of aromatic residues. The amide B double bands were observed at 2938 cm^−1^ and 2870 cm^−1^ corresponding to the two stretching modes of CH_2_. Stretching of C=O bonds of the polypeptide backbone was indicated by the presence of the amide I band at 1628cm^−1^. Amide II and amide III bands at 1539 cm^−1^ and 1234cm^−1^ respectively were indicative of N-H in plane bending vibrations coupled with C-N and C-H stretching. The remaining signals were assigned as follows: 1447 cm^−1^ O-H bending coupled with C-H scissoring, 1395 cm^−1^ carboxyl O-H bending, broad 1078 cm^−1^–1030 cm^−1^ double-band C-O stretching.

Contact angle analysis was carried out to evaluate the wettability of the hydrogel surface (Figure 3G). Water placed on control hydrogels showed significantly greater contact angle (mean, 29.03 ± 1.72) than water placed on treated hydrogels. Only the contact angle on non-treated PGTA 3 hydrogel was comparable to 3-SM hydrogel. The lowest contact angle was found on 3-SB hydrogels (mean, 7.76 ± 1.06), and was significantly different from the control hydrogel (*p* < 0.0001).

### 3.3. In Vitro Biocompatibility

**Figure 4:**
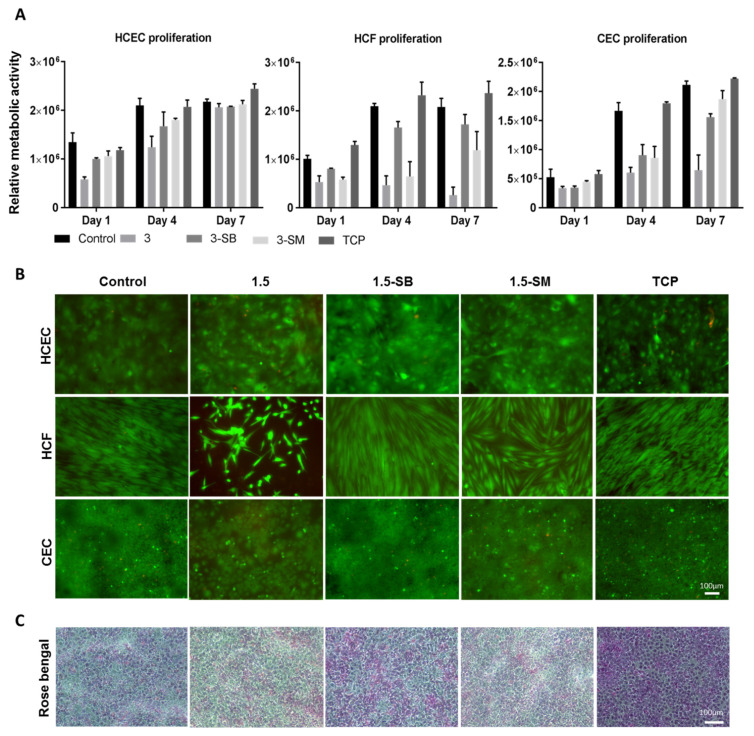
Biocompatibility studies of hydrogels 3 with three corneal cell types. (**A**) Metabolic activity study with individual cells at different time points, compared between hydrogels, with tissue culture plate (TCP) as a positive control. Quantitative results were reported as the mean ± S.D. (arbitrary unit) from three independent samples at each time points. (**B**) Live/dead staining of human corneal epithelial cells (HCEC), human corneal fibroblasts (HCF) and human corneal endothelial cells (CEC) on PFGT 3 hydrogels before and after post-crosslinked chemical treatment, compared with control hydrogel. All the images were taken at 7 days of cell culture. (**C**) Rose Bengal assay showed a normal pattern of stratification of the corneal epithelial cells, exhibiting multiple non-stained areas where the stratified epithelial barrier function excludes the dye. Scale bars are 100 μm.

The three major corneal cell types, HCEC, HCF, and CEC, were used to evaluate the biocompatibility of the hydrogels. Post-chemical treated 3 hydrogels were used for this experiment and unmodified hydrogels and TCP were used as controls. AlamarBlue assay was performed to evaluate cell metabolic activity (Figure 4A) and a live-dead assay (Figure 4B) was done to evaluate cytotoxicity related to hydrogel treatments. AlamarBlue assay showed that non-chemical treated 3 hydrogels were not biocompatible for any of the 3 corneal cells types, and this was confirmed by cytotoxicity testing. Sodium metabisulfite and sodium borohydride both prominently improved biocompatibility of the PFGT 3 hydrogel and facilitated the growth of corneal cells. Cell growth was better on the control non-treated hydrogels and on TCP than on to PFGT 3 hydrogels. 

The HCEC metabolic activity was superior on control hydrogels and the lowest cell growth was observed on PFGT 3 hydrogels at days 1 to 4. However, the metabolic activity of HCEC on all hydrogels became similar at day 7, showing non-significant differences (*p* = 0.1855). Live-dead staining data were similar. In the Rose Bengal assay, all the hydrogels showed a normal pattern of corneal epithelial cell stratification, with multiple non-stained areas where differentiated surface epithelial cells excluded the dye (Figure 4C).

HCF metabolic activity differences were prominent from day 4 of the cell culture. 3-SB hydrogels facilitated the growth of HCF and there was no significant difference in metabolic activity at day 7 between control hydrogels and 3-SB hydrogels (*p* = 0.4273). HCF metabolic activity was significantly lower at day 7 on 3-SM hydrogels compared to control hydrogels (*p* = 0.0090). Live-dead staining revealed that HCF became confluent at day 7 on 3-SB hydrogels, similar to control hydrogels and TCP.

CEC grew on hydrogels treated with both chemicals, whereas non-chemical treated hydrogels were not CEC compatible. There was no significant difference of cell growth between 3-SB and 3-SM hydrogels at day 7 (*p* = 0.1175). However, the cell number was higher on 3-SM hydrogels, and similar to control hydrogels and TCP (*p* = 0.2682 and 0.0646, respectively). Live-dead staining showed confluence of CEC by day 7 on all hydrogels and on TCP, except for non-chemical treated PGTA 3 hydrogels. 

### 3.4. In Vitro Evaluation of Human Adaptive Immunity in Presence of the Hydrogels

**Figure 5:**
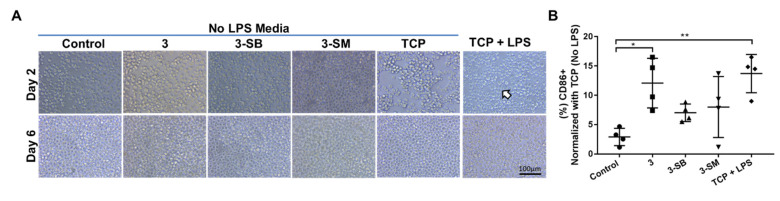
Human monocytic THP-1 cell polarization to M1 macrophage phenotypes in presence or absence of hydrogels and LPS. (**A**) THP-1 cells were cultured on different hydrogels and on TCP for 6 days. Morphological changes of cultured cells in presence of LPS are marked with arrow. (**B**) At day 6, the expression of CD86 (pro-inflammatory M1 marker) was evaluated and compared between the hydrogel groups, and percentage expression data was normalized to TCP (No LPS). Quantitative results were reported as the mean ± S.D. from four independent samples. * and ** represent *p* < 0.05, and *p* < 0.01, respectively.

We wanted to determine whether the monocytic cell line (THP-1) when cultured for 5 days on different PFGT 3 hydrogels would differentiate towards a pro-inflammatory M1 (CD86) macrophage phenotype. When LPS was not added in the media, no morphological changes were visible; however, when LPS was used in the media, the change in cell size and morphology were observed on TCP, which proved the differentiation potential of the THP-1 cells (Figure 5A). Expression of CD86 was evaluated after 6 days of culture on different hydrogels and TCP with or without LPS. Significant overexpression of CD86 (polarization toward M1 inflammatory macrophage) was noticed only on non-PFGT 3 hydrogels compare to the control (*p* = 0.0109). CD86 expression did not differ significantly between 3-SB and 3-SM hydrogels and the control (Figure 5B).

## 4. Discussion

The present study compared the effects of crosslinking collagen with two robust crosslinking agents, EDC and GTA. The results support a novel strategy to reduce the cytotoxic effect of the crosslinkers while strengthening the mechanical properties of the biomaterial, all without compromising the optical properties and enzymatic stability of the implant. Although EDC crosslinking did not show direct cytotoxic effects, GTA crosslinking of EDC crosslinked hydrogels induced cytotoxic effects. To address this concern, we restored cell biocompatibility by treating the double EDC/GTA crosslinked hydrogel with different chemicals (SB or SM).

There is a shortage of donor corneas to treat visual impairment due to corneal diseases, resulting in 10 million untreated patients with 1.5 million additional patients needing a transplant every year [25,26]. Only 1 donor cornea is available for 70 needed [27]. Hence, developing alternatives to human corneal donation is an urgent need. Crosslinked artificial corneas have been considered as a potential alternative to human donor corneas for transplantation, and have been shown in clinical trials to restore vision [3,28]. For the most part, collagen in these artificial corneas has been crosslinked with EDC and NHS. The EDC/NHS crosslinked hydrogel is mechanically weak and susceptible to enzymatic degradation. Therefore, there is an unmet need to generate stable collagen-based biomaterials. We have previously shown that double-crosslinking of collagen can be achieved by using EDC together with a bi-functional epoxy-based cross-linker, 1,4-butanediol diglycidyl ether [18]. We also demonstrated that this improves the elasticity and tensile strength of the collagen implants. In the current report, we have used two crosslinkers, EDC and GTA, to take advantage of the beneficial effects of each but at minimal concentrations. We show that this strategy reduces toxicity, and at the same time increases the mechanical and enzymatic stability of the hydrogels. The base hydrogel was made with collagen crosslinked with EDC/NHS, where the molar ratio EDC:Collagen-NH_2_ (mol:mol) = 0.7:1. EDC:Collagen-NH_2_ = 0.5–0.7 : 1 have been studied extensively as artificial corneas, and some formulations that have been transplanted into human patients [28,29]. Variation in the molar ratio of EDC:Collagen-NH_2_ was based on the type of collagen, its source, and the purpose of the study. Increased ratio of EDC can be used but will produce nonhomogenous hydrogels as the EDC becomes gelatinous very quickly and will not allow formation of particular organ structures such as the cornea. In our work, the hydrogels were treated with different concentrations of GTA for 4 h. In vitro biocompatibility studies of biomaterials with only GTA have shown that crosslinking with GTA for 24 h is not well-tolerated by human corneal epithelial cells [30]. In our study, we only used one time point, and we are aware that incubation for different time points could have produced different outcomes; it was shown that longer reaction times or higher GTA concentrations results in a decrease in free amine groups in the reaction [31].

We also found that the lowest concentration (PFGT 1 hydrogels) of GTA significantly increased the mechanical properties and enzymatic stability of the hydrogel. This is predictable as GTA crosslinking of collagen takes place through a reaction of the aldehyde groups of GTA with the amine groups of lysine or hydroxylysine residues [31]. While calculating the EDC concentration to make the hydrogel, we left unreacted lysine residues to react with GTA for secondary crosslinking. These conditions were gradually increased with increased concentrations of GTA, but at some point, increasing the GTA concentration (PFGT 5 hydrogels) did not alter the properties of the hydrogels, possibly due to saturation of the lysine group on the hydrogel. Cheung et al. proposed another explanation. They showed that lower concentrations of GTA were more effective in tissue crosslinking compared to higher concentrations, as high concentrations of GTA promote rapid surface crosslinking of the tissue, generating a barrier that prevents the diffusion of GTA into the tissue [32]. It was also shown that with high concentrations of GTA, the arrangement of collagen fibrils became very compact. Therefore, although the GTA dosage was increased, there was only a relatively small improvement in thermal stability and resistance to collagenase [13]. This was confirmed by our collagenase study in which PFGT 3 hydrogels exhibited maximum stability against collagenase, and that addition of more GTA (PFGT 5 hydrogels) did not render the hydrogel more stable. Our results parallel the previously published report that GTA-treated amniotic membrane is resistant to enzymatic digestion. Results from this latter study showed that the crosslinked membrane was preserved for up to 90 days without any signs of dissolution and maintained good transparency [33]. In our study, collagen gels crosslinked with GTA showed a yellowish color, which might be attributed to the self-polymerization of GTA molecules [13,34].

The challenging part of this project was to make the hydrogel biocompatible. For better GTA crosslinking, the reactions were carried out at neutral pH which induced the formation of reactive polymers. It has been suggested that the cytotoxicity and calcification arise from the propensity of GTA to form reactive polymers [35]. When human endothelial cells were seeded on untreated GTA-fixed aortic wall pieces, only limited adhesion (24%) was seen and no viable cells were found after 1 week [36]. On GTA-fixed heart valves, cell attachment was poor and no viable cells were observed [37]. Moreover, exposure time is also important. In vitro biocompatibility studies showed that the amniotic membranes (AM) crosslinked with GTA for 24 h do not support human corneal epithelial cell cultures, while AM treated with GTA for 6 h facilitated the expansion and transplantation of limbal epithelial progenitor cells [30]. Other research groups have showed that aldehyde groups introduced in the crosslinked biopolymers treated with GTA can be quenched with citric acid [36,37] and glycine [30,38] to reduce cytotoxicity. However, in our work we treated PFGT 3 hydrogels with citric acid, glycine, or lysine with no success as the toxicity of the hydrogels was not eliminated (data not shown). In contrast, the toxicity of GTA was reduced by reaction with sodium bisulfite via formation of a proposed GTA-bisulfite complex [39]. To our knowledge, neither SB nor SM has been used previously on GTA crosslinked biomaterials to improve biocompatibility. Another quenching agent we used was sodium borohydride. SB has been used as an aldehyde blocking reagent for electron microscope histochemistry [40] and for quenching of GTA-induced fluorescence in immunofluorescence on tissue sections [41].

Treatment with SB and SM had no adverse effect on the mechanical properties of double-crosslinked hydrogels. Enzymatic stability was similar before and after SB or SM treatment. Moreover, our studies confirmed that post-chemical treatment with SB or SM on double-crosslinked hydrogels rendered them optically clear and biocompatible. SB treated-hydrogels were similar to the control hydrogel in regard to transparency. The treatment with SB and SM will reduce aldehyde group and introduce the more hydrophilic hydroxyl or sulphate group and hence the product becomes more transparent. As the cornea is the main refractive element of the eye and serves as the main ocular diopter to transmit light for vision, high optical clarity is a key property that needs to be replicated in any artificial replacement [42]. The water content of human cornea is 80% and that of the collagen hydrogels was around 90% [18]. By FTIR spectra analysis of hydrated samples, we observed the typical collagen bands such as amide A at ~3310 cm^−1^, amide I at 1600–1700 cm^−1^, amide II at 1500–1550 cm^−1^, and amide III at 1200–1300 cm^−1^ [43]; however, amide B at ~3063 cm^−1^ corresponding to the collagen was missing. When dried sample was analyzed, amide B was observed and all bands shifted to the same degree across all the hydrogels. Shifting of bands is correlated with the degree of crosslinking [43] and in our case the modification of functional groups was similar for control and PFGT hydrogels. When GTA reacts with the lysine residues of proteins, the aldimine linkage (CH=N) forms, which has the characteristic absorption at 1450 cm^−1^ [44]. This band was missing even for non-PFGT hydrogels, which may be because of the minute modification of the lysine groups after double-crosslinking, although this slight modification contributed significantly to change the properties of the hydrogels. The contact angle significantly decreased after GTA crosslinking, resulting in more hydrophilic surfaces. This type of surface facilitates cell adherence and migration, which could lead to a rapid cellularization of the scaffold.

Immune cells, particularly monocytes and macrophages, play a critical role in determining success or failure of implant acceptance by the recipient [45]. Therefore, controlling macrophage polarity is one approach to control inflammation and prevent failure of implanted biomaterials [46]. Human monocytic THP-1 cells have been previously used to evaluate M1 macrophage differentiation in response to biomaterials [46,47]. We used THP-1 cells and monitored the expression of CD86 to determine the response to differently treated hydrogels. As macrophages are classically activated in vitro by bacterial cell wall components [48], we also used LPS as control for the differentiation of these cells on TCP. In general, reduced biocompatibility is associated with increased CD86 (M1 macrophage marker) expression [47]. We found that CD86 expression increased after GTA crosslinking in PFGT 3 hydrogels. However, treatment of hydrogels with SB or SM mitigated the overexpression of CD86.

## 5. Conclusions

Our results demonstrate that double-crosslinking improves the mechanical properties of the scaffolds, and the treatment with SB or SM improves biocompatibility. This unique developmental approach should facilitate the use of collagen-based implants in regenerative medicine.

## Figures and Tables

**Table 1:** Antibodies for Flow Cytometry.

Target	Antibody	Supplier	Dilution Factor
CD86	APC Mouse Anti-Human CD86, Clone 2331 (FUN-1)	BD Bioscience, Odenton, MD, USA	1/20
Isotype Control for CD86	APC Mouse IgG1, κ, Clone MOPC-21	BD Bioscience	1/20

## Data Availability

The datasets generated during and/or analysed during the current study are available from the corresponding author on reasonable request.
